# An efficient algorithm to explore liquid association on a genome-wide scale

**DOI:** 10.1186/s12859-014-0371-5

**Published:** 2014-11-28

**Authors:** Tina Gunderson, Yen-Yi Ho

**Affiliations:** Division of Biostatistics, School of Public Health, University of Minnesota, 420 Delaware St. S.E., MMC 303, Minneapolis, 55455 MN USA

**Keywords:** Coexpression pattern, Liquid association, Genome-wide search

## Abstract

**Background:**

The growing wealth of public available gene expression data has made the systemic studies of how genes interact in a cell become more feasible. Liquid association (LA) describes the extent to which coexpression of two genes may vary based on the expression level of a third gene (the controller gene). However, genome-wide application has been difficult and resource-intensive. We propose a new screening algorithm for more efficient processing of LA estimation on a genome-wide scale and apply its use to a *Saccharomyces cerevisiae* data set.

**Results:**

On a test subset of the data, the fast screening algorithm achieved >99.8*%* agreement with the exhaustive search of LA values, while reduced run time by 81–93 *%*. Using a well-known yeast cell-cycle data set with 6,178 genes, we identified triplet combinations with significantly large LA values. In an exploratory gene set enrichment analysis, the top terms for the controller genes in these triplets with large LA values are involved in some of the most fundamental processes in yeast such as energy regulation, transportation, and sporulation.

**Conclusion:**

In summary, in this paper we propose a novel, efficient algorithm to explore LA on a genome-wide scale and identified triplets of interest in cell cycle pathways using the proposed method in a yeast data set. A software package named **fastLiquidAssociation** for implementing the algorithm is available through http://www.bioconductor.org.

**Electronic supplementary material:**

The online version of this article (doi:10.1186/s12859-014-0371-5) contains supplementary material, which is available to authorized users.

## Background

Large-scale gene expression data provide snapshots of transcription activity at a genome-wide scale. There is a growing wealth of gene expression data available in public databases (such as the Gene Expression Omnibus) and as well as the capability for easily generating additional data using high-throughput technologies.

Many methods for the statistical analysis of gene expression data exist [1]. Initially data analyses for differential expression focuse on a single gene at a time [2-4]. These one-gene-at-a-time analyses separate data into groups depending on the phenotypic status and perform gene-by-gene analysis. However recently the focus has shifted to higher order coexpression patterns (i.e. correlations of the expression levels of two or more genes) with the belief that they may reflect more fully the complex interactions between genes [5-11].

One type of multi-dimensional differential expression analysis is called liquid association. Liquid association (LA) describes the extent to which coexpression of two genes (*X*_1_,*X*_2_) may vary based on the expression level of a third gene (*X*_3_), with the third gene being viewed as a controller gene that can represent the pathway status or the cellular state [7]. Liquid association has been demonstrated to be useful in identifying disease candidate genes for multiple sclerosis and performing dimension reduction for candidate genes in survival studies [12,13]. Li’s work [7] applied LA in two distinct ways. The first fixed a controller gene (i.e. the gene in the *X*_3_ position) or a small subset of controller genes and searched for pairs of genes (*X*_1_,*X*_2_) that showed significant liquid association while the second method reversed this process, specifying one or both of the pair of genes (*X*_1_,*X*_2_) and searching for a controller gene (*X*_3_) that regulates their correlation [7,8,12].

Software is available to assist in the calculation of individual liquid association triplets as in Li’s work, and one study has performed brute-force exhaustive searches for liquid association [14]; however neither of these approaches are efficient for genome-wide use. Computational analyses for LA on a genome-wide scale have proven more intractable due to the issue of dimensionality, with the number of possible combinations increasing exponentially in a situation where the number of samples is already greatly exceeded by the number of genes potentially of interest. For example, in a typical microarray with 6,000 genes, there are more than 1.079×10^11^ all possible triplet combinations need to be examined in a exhaustive search. In other words, assuming each LA calculation took one one-thousandth of a second, the full calculation of all possible values when performed in sequence would still take approximately 3.4 years. Obviously a different approach is needed. Thus in this paper, we develop a fast-screening algorithm with an R software package available for applying liquid association in a genome-wide scale search and implement it in a yeast data set.

## Methods

### Data set

We used the yeast dataset described in [15]. Yeast is a model organism for studying complex gene interdependencies due to its short generation time, ease of culture, and that yeast’s fundamental biological processes are conserved among all eukaryotes, which allows us to apply the increased understanding obtained to other organisms [16]. The raw data set is publically available at the Yeast Cell Cycle Analysis Project website and was also available in [15]. The data set contains the gene expression measures for 6,178 yeast genes under 73 normal growth conditions and was intended to represent a comprehensive catalog for transcripts that vary periodically within the cell cycle [15].

### Methods for estimating liquid association

Li [7] used *E*(*X*_1_*X*_2_|*X*_3_) to measure the co-expression of *X*_1_ and *X*_2_, and ultimately results in an estimation of *LA*(*X*_1_,*X*_2_|*X*_3_)=*E*(*X*_1_*X*_2_*X*_3_), with the standard error obtainable by bootstrap [7]. Ho et al. [17] noted that Li’s measure does not account for instances where the conditional means and variances of *X*_1_ and *X*_2_ may depend on *X*_3_ and proposed a new measure named modified liquid association (MLA). Compared to Li’s original measure, MLA is able to consider more intricate co-dependencies among these variables and was proven to be more robust for data analysis applications [17]. Hence in the following analysis, we applied MLA to assess the magnitude of liquid association.

To estimate MLA, both a robust direct estimate and a trivariate conditional normal model (CNM) framework were proposed in [17]. For instances where the CNM does not fit the data well, the more robust direct estimate with bootstrapping standard error can be used.

**Figure 1:**
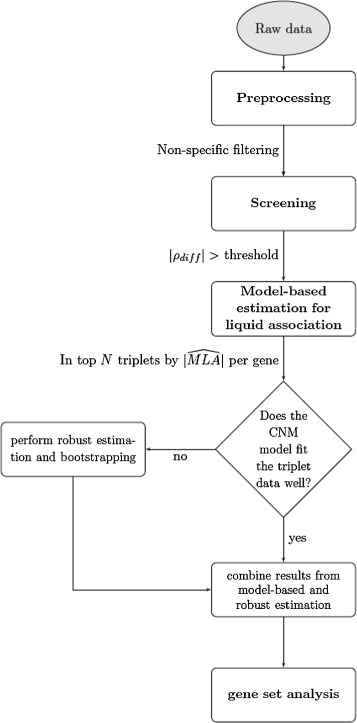
**A process map of the fastLA algorithm.**

The focus of the paper is to develop a screening algorithm which would make it faster to perform a genome-wide analysis of a data set to check for evidence of dependent coexpression. Our algorithm named fast liquid association (**fastLA**) seeks to reduce the number of triplets needing to be examined in depth in two steps: (1) screening and (2) model fitting and estimation. As illustrated in Figure 1, after proper preprocessing, in the screening step triplets unlikely to have a significant LA value were removed. The screening step relies on the |*ρ*_*diff*_| score, with *ρ*_*diff*_ defined as:

(1) $$ \rho_{diff}=\rho_{high} - \rho_{low},  $$

where *ρ*_*high*_ is the Pearson correlation when the third controller gene (*X*_3_) is high (in the top tertile) and *ρ*_*low*_ is the correlation when *X*_3_ is low (in the bottom tertile). *ρ*_*diff*_ is a suitable screening measure for liquid association for the following reasons: (1) in the situation when *X*_3_ is discretized into three values: −1,0,1 by tertile binning, *ρ*_*diff*_ equals to the liquid association measure by definition:

$$\begin{array}{@{}rcl@{}} LA&=& \frac{\text{change in coexpression}}{\text{change in \(X_{3}\)}} = \frac{\text{change in } \rho}{\text{change in } X_{3}} \\ &=& \frac{\rho_{high}-\rho_{medium}}{1-0} \\ &&+ \frac{\rho_{medium}-\rho_{low}}{0-(-1)} = \rho_{high} - \rho_{low}, \\ \end{array} $$

where *ρ*_*median*_ is the correlation when *X*_3_ is in the middle tertile. Triplet combinations that exhibit large *ρ*_*diff*_ value are likely to manifest large liquid association. (2) *ρ*_*diff*_ can be computed much more quickly through matrix algebra than MLA estimation.

After the first screening step, triplet combinations with a large |*ρ*_*diff*_| value were retained for further model fitting and estimation. As illustrated in Figure 1, during the second step of the algorithm, the magnitude of liquid association is estimated through the CNM if the model fits the triplet data well. Two versions for estimating MLA using the CNM are available, a full and simple version of the model, depending on which model fits the triplet data better. In the case when the CNM model does not adequately describe the data, the robust estimation can be used instead. More detail about the CNM and robust estimation procedure are described in [17]. Gene set enrichment analysis using Gene Ontology [18] were performed for the top triplet combinations identified in the yeast dataset [15].

## Results

### Validation

**Figure 2:**
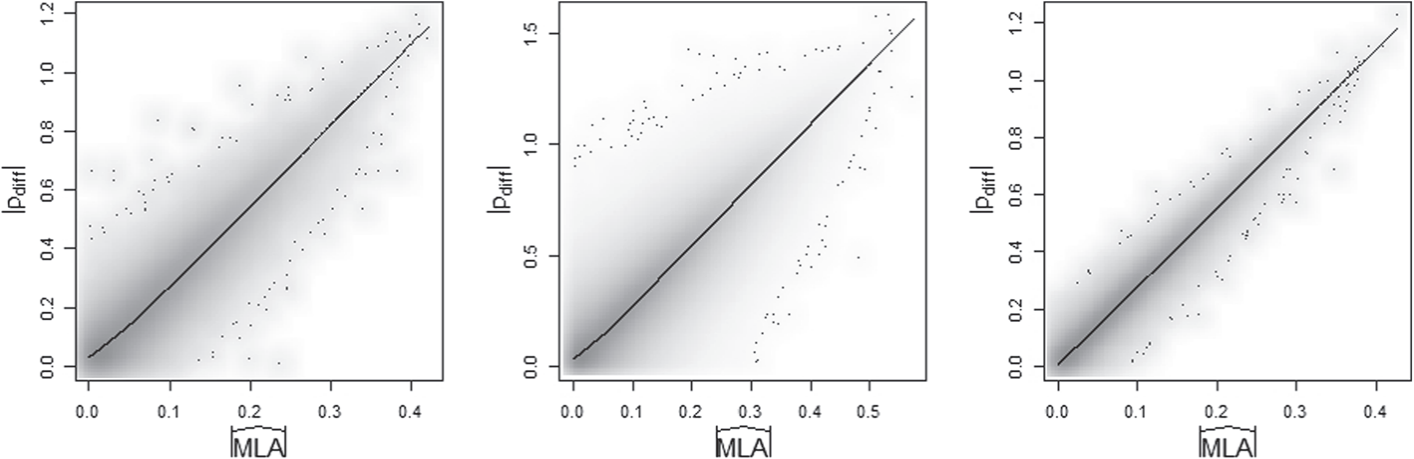
**Comparison for all triplets of** $|\widehat {MLA}|$ **vs. |** ***ρ***_***diff***_ **|.** The plot for 50 gene subset is on the left, 250 gene subset in the middle, and simulated data from multivariate normal distribution with mean 0 and identify variance-covariance matrix on the right.

Similar to the approach applied by both Li [7] and Ho et al. [17], we first performed a normal quantile transformation of the data so that marginally each variable was normally distributed. This approach could also help to reduce the number of potential outliers in the data. In addition, each gene was also standardized to have mean 0 and variance 1. We removed any genes with greater than 30 *%* missing values. This reduced the number of genes being tested to 5,721. We randomly pick 50 genes and 250 genes from the yeast data set to determine agreement between *ρ*_*diff*_ and liquid association estimates $\left (\widehat {MLA}\right)$. The results are shown in Figure 2; in the plot on the left, the correlation between *ρ*_*diff*_ and $\widehat {MLA}$ is 0.968 in the 50 gene subset; 0.960 in the 250 gene subset as illustrated by the plot in the middle; 0.990 for simulated data from multivariate normal distribution with mean 0 and identify variance-covariance matrix on the right. When absolute values were not taken, there was 100 *%* agreement in sign. We performed simple linear regression: $|\rho _{\textit {diff}}| = \alpha + \beta *|\widehat {MLA}|$. Interestingly, the *β* estimates are approximately 2.69, 2.68, and 2.75 respectively in the 50 subset, 250 subset, and simulated data from multivariate normal distribution. This value compares well to the possible maximum values for |*ρ*_*diff*_| (2.0) and $|\widehat {MLA}|\left (\sqrt {2/\pi }\right)$ as $2/\sqrt {2/\pi }=2.507$.

**Figure 3:**
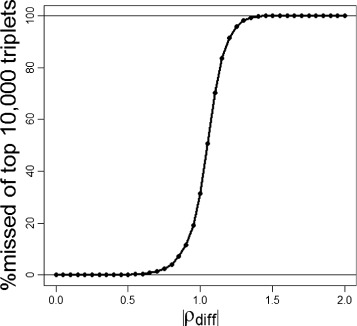
**The percent missed in the top** $|\widehat {MLA}|$ **10,000 triplets in the 250 gene subset by increasing values of |** ***ρ***_***diff***_ **|.**

Using the 250 genes subset, we performed the fastLA and an exhaustive analysis in order to perform a speed comparison as well as to test for sensitivity. Based on sensitivity analyses, the data was separated into three bins for the model-based estimate of MLA to minimize mean squared error according to Ho et al. [17]. Testing was performed at |*ρ*_*diff*_|=0.3 and 0.5. The proportion of the top $|\widehat {MLA}|$ 10,000 triplet sets found using fastLA versus those found using exhaustive liquid association analysis was >99*%* for both |*ρ*_*diff*_|=0.3 and 0.5 (at matches of 99.98% and 99.87% respectively). The proportion of the top $|\widehat {MLA}|$ 10,000 triplets missed by varying values of |*ρ*_*diff*_| are shown in Figure 3.

By narrowing the triplets with |*ρ*_*diff*_|>0.5, we reduced the number of the triplet combinations needed to be examined from 7,719,000 to 918,688 triplets (11.9% of all triplet combinations) in the 250 gene subset analysis. In the middle plot of Figure 2, only two out of 7,719,000 triplets (rank 80 and 2680 among all triplets) have |MLA|>0.4 (≈ 50% of maximum MLA value) and are missed by *ρ*_*diff*_>0.5 screening criteria. Because of discretizing *X*_3_, there is a small reduction of resolution for measuring MLA using *ρ*_*diff*_ in these two cases. However, the reduction in run time was substantial due to a much smaller number of triplets needed to be examined after the screening. Compared to the exhaustive analysis, the relative run time required for completion using the fastLA algorithm was 19.1% when using |*ρ*_*diff*_|=0.3 as the cut-off threshold and 6.51% when using |*ρ*_*diff*_|=0.5 (run times 2876 seconds and 979 seconds respectively vs. 15046 seconds using the exhaustive search). Processing was performed on servers at the Minnesota Supercomputing Institute on two-socket, quad-core 2.8 GHz Intel Xeon X5560 Nehalem EP processors with 22 GB of RAM.

We set |*ρ*_*diff*_|=0.5 and implemented the fastLA algorithm in the yeast dataset. After the first screening step, 1.179×10^10^ (12.6%) triplets out of 9.357×10^10^ triplets remained in the second step. The results were sorted using the model-based estimation for liquid association. The top 10 triplet combinations are shown in Table 1 sorted by p-value [19], and a fuller list of the top 10,000 triplets is presented in the Additional file 1. In Table 1, the model column represents the way the p-value was derived (F = results from full CNM model, S = results from simple CNM model). For genes where the function is characterized, the RefSeq gene symbol is reported. For those genes whose function has not yet been characterized, the open reading frame ID was reported instead. In addition, we analyzed the data separately by four synchronization conditions in which the yeast experiments were performed. The box plots of gene expression, and the top 100 triplets with large MLA values in each synchronization condition are provided in the Additional files 2, 3, 4, 5, and 6 respectively.

**Table 1:** **Top 10 triplets by p-value**

	***X***_**1**_ **/** ***X***_**2**_	***X***_**2**_ **/** ***X***_**1**_	***X***_**3**_	***ρ***_***diff***_	${\hat {MLA}}$	**Wald**	**p-value**	**p-adj**	**Model**
1	SKN1	GAS2	YGR149W	1.335	0.417	49.050	2.501E-12	5.332E-05	F
2	YCRX13W	YFL052W	STL1	1.234	0.417	47.600	5.217E-12	5.332E-05	F
3	UBC5	RTG2	MLH2	-1.325	-0.471	46.720	8.194E-12	5.332E-05	F
4	RSM28	YLR281C	PLB1	1.042	0.405	46.470	9.290E-12	5.332E-05	F
5	SRO77	SNQ2	TFC3	-1.029	-0.406	46.280	1.023E-11	5.332E-05	F
6	YIM2	THI80	MUP1	1.204	0.437	46.110	1.118E-11	5.332E-05	F
7	YIL169C	YJL193W	AIM45	1.171	0.431	45.250	1.737E-11	7.100E-05	F
8	SIZ1	MCM16	AXL1	1.368	0.449	43.700	3.826E-11	1.368E-04	F
9	PYC2	HKR1	YPR170C	-1.199	-0.434	43.390	4.489E-11	1.427E-04	F
10	SEO1	YPL113C	RSC4	-1.162	-0.410	43.030	5.389E-11	1.541E-04	F

In *saccharomyces cerevisiae*, there are 171 genes with transcription factor specificities that show DNA binding ability and have at least 1 identified motif according to the yeast transcription factor compendium [20]. In the top 342 triplet combination with p value <10^−8^, 10 (5.8%) of the 171 genes were reported as the controller gene (*X*_3_) in the list. These 10 genes are provided in Additional file 7.

### Results of GO analysis

We performed gene set enrichment analysis using GO [18] for the 342 triplet combinations with p value <10^−^8, both for the genes in the *X*_3_ position (328 unique genes) and for all genes in the triplets (905 unique genes) using a significance level *α*=0.05 for the analyses. The conditional Fisher’s exact test was used to account for the hierarchical structures in GO. We reported the top 15 GO terms using biological process ontology in Table 2 and 3. The full list of enriched GO terms in biological process, molecular function and cellular component ontology are reported in the Additional files 8, 9, 10, 11, 12, and 13. Pathways composed of fewer than five genes are not reported in the analysis.

**Table 2:** **Top 15 GO terms for** ***X***_**3**_ **analysis using biological processes ontology**

	**GOBPID**	**Pvalue**	**OddsRatio**	**ExpCount**	**Count**	**Size**	**Term**
1	GO:1901137	6.38E-03	1.94	11.74	21	208	Carbohydrate derivative biosynthetic process
2	GO:0016072	6.75E-03	1.87	13.32	23	236	rRNA metabolic process
3	GO:0005979	8.07E-03	10.12	0.45	3	8	Regulation of glycogen biosynthetic process
4	GO:0018202	8.07E-03	10.12	0.45	3	8	Peptidyl-histidine modification
5	GO:0051180	8.07E-03	10.12	0.45	3	8	Vitamin transport
6	GO:0001402	1.16E-02	8.43	0.51	3	9	Signal transduction involved in filamentous growth
7	GO:0015986	1.16E-02	8.43	0.51	3	9	ATP synthesis coupled proton transport
8	GO:0032885	1.16E-02	8.43	0.51	3	9	Regulation of polysaccharide biosynthetic process
9	GO:0072348	1.97E-02	4.50	1.07	4	19	Sulfur compound transport
10	GO:0043269	2.09E-02	6.32	0.62	3	11	Regulation of ion transport
11	GO:0006506	2.15E-02	3.52	1.64	5	29	GPI anchor biosynthetic process
12	GO:0009303	2.15E-02	3.52	1.64	5	29	rRNA transcription
13	GO:0000462	2.21E-02	2.24	4.86	10	86	Maturation of SSU-rRNA from tricistronic rRNA transcript (SSU-rRNA, 5.8S rRNA, LSU-rRNA)
14	GO:0016579	2.35E-02	4.22	1.13	4	20	Protein deubiquitination
15	GO:0000479	2.61E-02	2.90	2.31	6	41	Endonucleolytic cleavage of tricistronic rRNA transcript (SSU-rRNA, 5.8S rRNA, LSU-rRNA)

**Table 3:** **Top 15 GO terms for full triplet analysis using biological processes ontology**

	**GOBPID**	**p-value**	**OddsRatio**	**ExpCount**	**Count**	**Size**	**Term**
1	GO:0006335	2.72E-03	21.39	0.79	4	5	DNA replication-dependent nucleosome assembly
2	GO:0006096	4.37E-03	3.11	4.74	11	30	Glycolysis
3	GO:0009071	7.13E-03	10.69	0.95	4	6	Serine family amino acid catabolic process
4	GO:0000266	1.46E-02	7.13	1.11	4	7	Mitochondrial fission
5	GO:0015677	1.46E-02	7.13	1.11	4	7	Copper ion import
6	GO:0042938	1.46E-02	7.13	1.11	4	7	Dipeptide transport
7	GO:0009205	1.95E-02	1.59	21.51	31	136	Purine ribonucleoside triphosphate metabolic process
8	GO:0071470	2.18E-02	2.44	5.06	10	32	Cellular response to osmotic stress
9	GO:0001079	2.55E-02	5.34	1.27	4	8	Nitrogen catabolite regulation of transcription from RNA polymerase II promoter
10	GO:0051180	2.55E-02	5.34	1.27	4	8	Vitamin transport
11	GO:0006184	2.82E-02	1.76	12.18	19	77	GTP catabolic process
12	GO:0006446	2.84E-02	2.88	3.16	7	20	Regulation of translational initiation
13	GO:0032889	2.94E-02	3.82	1.90	5	12	Regulation of vacuole fusion, non-autophagic
14	GO:0000154	2.97E-02	3.21	2.53	6	16	rRNA modification
15	GO:0006000	3.07E-02	8.01	0.79	3	5	Fructose metabolic process

Given that the Spellmen et al. experiments created nutrient-depleted conditions for growth, it is biologically feasible to see that for the controller position (*X*_3_), many top terms are involved energy regulation such as carbohydrate derivative, glycogen, and polysaccharide biosynthesis described in Table 2. Glycogen in yeast is formed during periods where carbon, nitrogen, phosphorus or sulfur is limited [21]. In addition, several top terms are related to transportation of cellular molecules such as vitamin, sulfur compound, and ion. Furthermore, GPI-anchor protein biosynthesis could be related to cell wall formation for sporulation during nutrient-depleted environment.

The results from the analyses using the full triplet set trend toward functions of energy regulation, and transport of molecules as shown in Table 3. Glycolysis is related to the utilization of glucose. In addition, regulation of lipid, carbohydrate, hexose, purine ribonucleotide could also be involved in energy regulation process. The findings presented by GO analysis could suggest feasible biological hypotheses; however, liquid association measure describes ‘association” between gene triplet, but it does not necessary confers “causation.” Further functional experiments will be needed to validate the top triplets identified with large MLA values.

## Discussion

In the data analysis, we set |*ρ*_*diff*_|=0.5. There are a few considerations about setting the threshold value for |*ρ*_*diff*_|. The maximum value is theoretically 2 (as *ρ*_*diff*_=*ρ*_*high*_−*ρ*_*low*_ and $-1 \le \rho _{X_{1},X_{2}} \le 1$). For general use, too high a value for |*ρ*_*diff*_| risks missing those triplets whose MLA values are not fully reflected by the more simplistic correlation, while too low a value approaches testing all possible triplets and forfeits any increase in testing efficiency. We set the default value at 0.5 (25% of the realizable correlation difference) as we found >99.98*%* of the triplets with a large MLA were captured by setting |*ρ*_*diff*_|=0.5 in the validation subset. If we increase the threshold for the |*ρ*_*diff*_| cutoff, we could further decrease time without substantial loss in sensitivity. Of the top 10,000 triplets, only 128 would have been missed using a cutoff of |*ρ*_*diff*_|=1.0. However, this would have substantially decreased the number of triplets that needed to be checked for MLA estimates, which in turn would have helped decrease memory usage and overall processing time.

In the algorithm, *ρ*_*diff*_ is calculated based on the difference between a “high” versus “low” subset of the data for each gene in the controller position. Initially the median (after removal of any data with a missing value in the *X*_3_ position) was used as the demarcation between the high and low subsets. However, we found that the central points diluted the *ρ*_*diff*_ estimate and decreased sensitivity. The algorithm was respecified to split the data into three parts based on the *X*_3_ values, with high being the top third and low being the bottom third in our analysis. By using the upper and lower tertiles for Z expression, the values of *ρ*_*diff*_ increase in triplets with large liquid association and hence increase the sensitivity to identify triplets with large MLA values. Based on data obtained in the verification process, we used this specification of the algorithm in this analysis. Furthermore, the splitting of *X*_3_ can be easily modified for other analysis; however, in practice, we suggest to have between 15 to 30 samples as recommended by Ho et al. [17] samples in each bin to achieve stable estimates of *ρ*.

During the course of parameter estimations using the CNM models, we identified a subset of triplets where the CNM does not fit the data well. In total, of the 2.8605×10^7^ triplets that were tested using the full model, 23,830 triplets can not be adequately described by the CNM full model.

**Figure 4:**
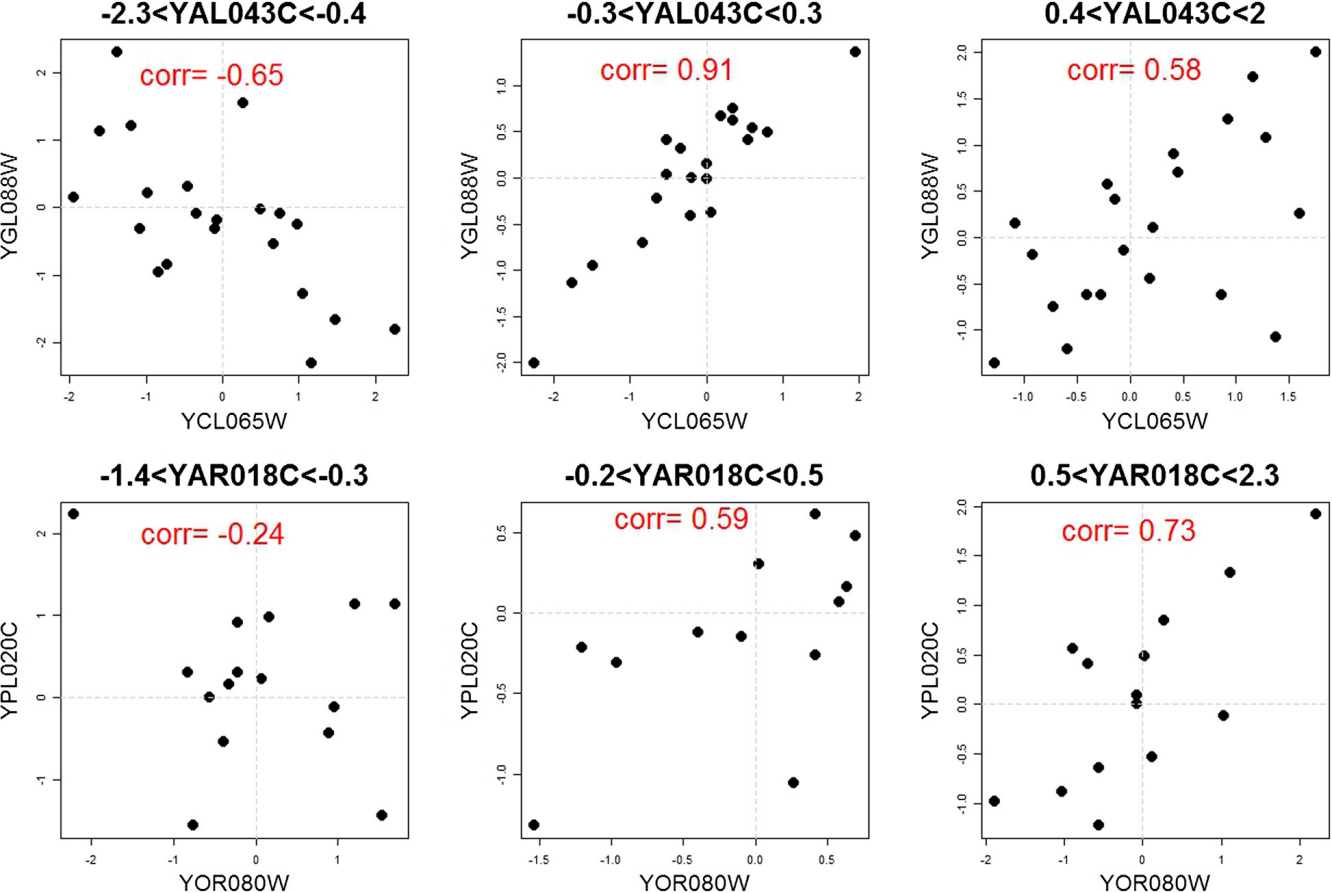
**Triplets with lack of fit to the conditional normal model (CNM).** The *X*_1_,*X*_2_ genes form the axes and the title lists the *X*_3_ gene.

Of these 23,830 triplets, 21,935 (92.0%) triplets were estimated using the simple CNM model, and 1,895 (7.95%) were estimated using the robust method. After investigating these triplets, we identified the following possible explanations for why they are not appropriately fit by the CNM: (1) The distribution of *X*_1_,*X*_2_ is not bivariate normal with respect to *X*_3_, (2) The change in $\rho _{X_{1}X_{2}|X_{3}}$ is non-linear with respect to *X*_3_, or (3) The model’s reliance on Pearson correlation makes it more sensitive to outliers. None of the triplets tested using the direct estimate method were found to be of sufficiently low adjusted p-value to be included in the set of the top gene triplet combinations. Figure 4 provides example scatter plots of triplets that do not fit the CNM well. The first row is an example of non-linear changes in correlation with regards to the value of the *X*_3_ gene. The second row provides an illustration of the bins’ susceptibility to outliers, in the that correlation for both the leftmost and center plots would be changed without the single outlier on the left. In these cases, the robust estimation procedure is more appropriate to assess the magnitude of liquid association.

A concern that has been raised in regards to using Hypergeometric-based tests is the problem of defining the gene universe. When a larger gene universe is used, it in general will tend to (assuming all other variables remain the same) have the effect of making the p-value seem more significant [4]. Given the genome-wide scope and nature of our testing (in that a priori, we had no way of distinguishing which genes might be found to be “interesting” and thus all genes were equally likely to be selected), it was decided that all analyzed genes would be included in the gene universe for analysis and the results interpreted conservatively. While the data used from Spellman et al. were obtained from cDNA arrays and thus more likely to have prior rationale of biological plausibility for probe inclusion, for commercial chips performing some non-specific filtering prior to analysis may help reduce the size of the gene universe and manage to avoid the issue.

## Conclusion

We proposed the fastLA algorithm for exploring liquid association in a genome-wide scale. Some modifications of the fast liquid association algorithm could be: (1) For binary traits, *ρ*_*diff*_ can be used as the liquid association measure. Our algorithm can be easily adapted to the binary case, (2) Use a rank-based correlation statistic. Using non-parametric correlation would make the model more robust to outliers and potential violations of the assumption that the variables are bivariately normally distributed; however, rank-based correlation statistic could be less statistically powerful comparing to the Pearson correlation.

On the basis of the results of this study, it appears that *ρ*_*diff*_ would be an appropriate screening metric for MLA in use for exploratory genome-wide searches and that both metrics are suitable for identifying triplets of interest. Given the high correlation observed between *ρ*_*diff*_ and MLA and the increased speed of calculation of *ρ*_*diff*_ due to its matrix manipulation to perform the estimate, this would significantly reduce both processing time and memory requirements. While there remain reservations that *ρ*_*diff*_ may not be suitable for a comprehensive identification of triplets of significant p-values, nevertheless it is a fast and efficient screening tool to identify potentially significant gene triplets using liquid association.

## Additional files

Additional file 1
**A list of the top 10,000 triplets reported by the fastLA algorithm using the yeast data set.**

Additional file 2
**Box plots of gene expression measurements by four synchronization conditions.**

Additional file 3
**A list of the top 100 triplets reported by the fastLA algorithm using the yeast data set following pheromone-based synchronization.**

Additional file 4
**A list of the top 100 triplets reported by the fastLA algorithm using the yeast data set following cdc15-based synchronization.**

Additional file 5
**A list of the top 100 triplets reported by the fastLA algorithm using the yeast data set following cdc28-based synchronization.**

Additional file 6
**A list of the top 100 triplets reported by the fastLA algorithm using the yeast data set following elutriation synchronization.**

Additional file 7
**10 gene triplets among the top 342 triplets with p value < 10** ^**−**^ **8 that show transcription factor specificities.**

Additional file 8
**Enriched GO biological process categories for** ***X***_**3**_ **controller gene in the top triplets with significant MLA values.**

Additional file 9
**Enriched GO cellular component categories for** ***X***_**3**_ **controller gene in the top triplets with significant MLA values.**

Additional file 10
**Enriched GO molecular process categories for** ***X***_**3**_ **controller gene in the top triplets with significant MLA values.**

Additional file 11
**Enriched GO biological process categories for genes in the top triplets with significant MLA values.**

Additional file 12
**Enriched GO cellular component categories for genes in the top triplets with significant MLA values.**

Additional file 13
**Enriched GO molecular process categories for genes in the top triplets with significant MLA values.**
