# Supplementary figures and images for: An efficient algorithm to explore liquid association on a genome-wide scale
Source: BMC Bioinformatics. 2014 Nov 28;15(1):371. doi: 10.1186/s12859-014-0371-5 (PMC4255454; doi:10.1186/s12859-014-0371-5)

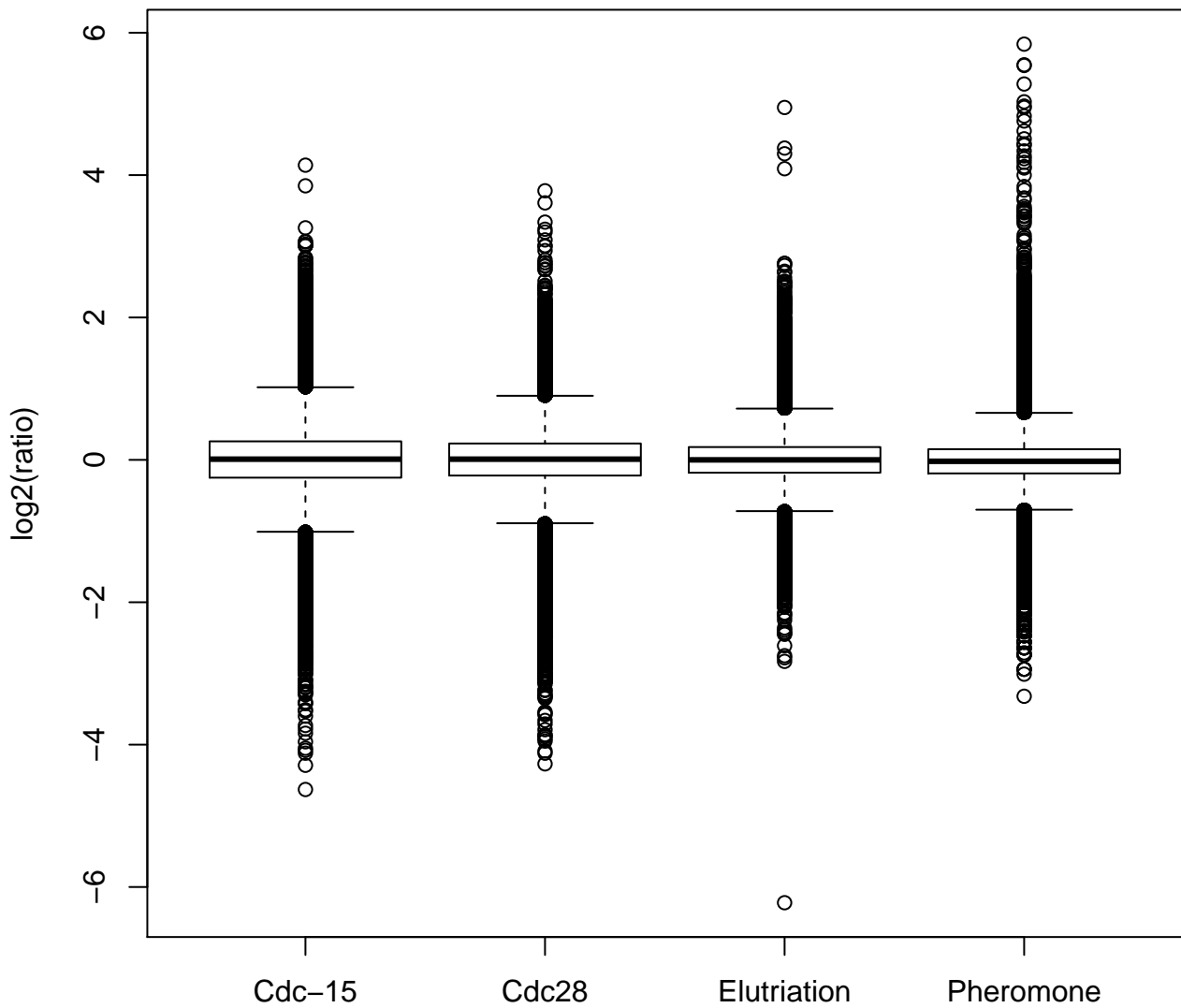

Supplement: Additional file 2 — Box plots of gene expression measurements by four synchronization conditions. [file 12859_2014_371_MOESM2_ESM.pdf]
